# Frontotemporal Transcranial Direct Current Stimulation Decreases Serum Mature Brain-Derived Neurotrophic Factor in Schizophrenia

**DOI:** 10.3390/brainsci11050662

**Published:** 2021-05-19

**Authors:** Ondine Adam, Marion Psomiades, Romain Rey, Nathalie Mandairon, Marie-Francoise Suaud-Chagny, Marine Mondino, Jerome Brunelin

**Affiliations:** 1 Centre Hospitalier Le Vinatier, F-69500 Bron, France; ondine.adam@ch-le-vinatier.fr (O.A.); romain.rey@ch-le-vinatier.fr (R.R.); nathalie.mandairon@cnrs.fr (N.M.); marine.mondino@ch-le-vinatier.fr (M.M.); 2 INSERM U1028, CNRS UMR5292, PSYR2 Team, Lyon Neuroscience Research Center, Université Claude Bernard Lyon 1, Université Jean Monnet, F-69500 Bron, France; marion.psomiades@ch-le-vinatier.fr (M.P.); marie-francoise.suaud-chagny@ch-le-vinatier.fr (M.-F.S.-C.); 3 INSERM U1028, CNRS UMR5292, NEUROPOP Team, Lyon Neuroscience Research Center, Université Claude Bernard Lyon 1, Université Jean Monnet, F-69500 Bron, France

**Keywords:** tDCS, schizophrenia, plasticity, mature BDNF

## Abstract

Although transcranial direct current stimulation (tDCS) shows promise as a treatment for auditory verbal hallucinations in patients with schizophrenia, mechanisms through which tDCS may induce beneficial effects remain unclear. Evidence points to the involvement of neuronal plasticity mechanisms that are underpinned, amongst others, by brain-derived neurotrophic factor (BDNF) in its two main forms: pro and mature peptides. Here, we aimed to investigate whether tDCS modulates neural plasticity by measuring the acute effects of tDCS on peripheral mature BDNF levels in patients with schizophrenia. Blood samples were collected in 24 patients with schizophrenia before and after they received a single session of either active (20 min, 2 mA, *n* = 13) or sham (*n* = 11) frontotemporal tDCS with the anode over the left prefrontal cortex and the cathode over the left temporoparietal junction. We compared the tDCS-induced changes in serum mature BDNF (mBDNF) levels adjusted for baseline values between the two groups. The results showed that active tDCS was associated with a significantly larger decrease in mBDNF levels (mean −20% ± standard deviation 14) than sham tDCS (−8% ± 21) (F = 5.387; *p* = 0.030; η^2^ = 0.205). Thus, mature BDNF may be involved in the beneficial effects of frontotemporal tDCS observed in patients with schizophrenia.

## 1. Introduction

Auditory verbal hallucinations (AVH) are disabling and frequent symptoms in patients with schizophrenia. Among the available therapeutic strategies, some evidence suggests that transcranial direct current stimulation (tDCS) with the anode placed over the left prefrontal cortex and the cathode placed over the left temporoparietal junction may alleviate treatment-resistant AVH [1] up to three months after tDCS treatment [2]. However, although tDCS has shown encouraging clinical results [3], the brain mechanisms through which tDCS may have sustainable beneficial effects on AVH remain unclear. During tDCS, a weak direct current is circulating between two electrodes placed over the scalp of the subject. Physiological effects of tDCS are thought to be mediated by a modulation of the cortical excitability of brain regions situated under the location of electrodes [4]. The effects do not seem limited to the targeted cortical area and the modulatory effect may also spread to a large network of inter-connected brain regions [5,6]. These local and regional effects can outlast the stimulation period, suggesting long-term potentiation (LTP)/long-term depression (LTD)-like mechanisms [7], which require activity-dependent brain-derived neurotrophic factor (BDNF) secretion [8,9].

BDNF is a neurotrophic factor involved in neuronal plasticity including neuronal growth, cortical excitability, and neuronal regeneration mechanisms [10]. Several lines of evidence from animal research have suggested that the effects of tDCS depend on endogenous BDNF levels [11], and that tDCS promotes LTP mechanisms by increasing the expression of BDNF [12]. However, clinical studies investigating the effects of tDCS on BDNF levels in patients with neuropsychiatric conditions have produced controversial results. Although some studies showed that tDCS can induce an increase in serum BDNF levels in opioid-addicted patients [13] or in patients with Parkinson’s disease [14], others did not detect any effects of tDCS on BDNF levels in patients with major depressive disorder [15,16] and bipolar disorder [17]. To the best of our knowledge, no studies have investigated the effect of tDCS on peripheral BDNF levels in patients with schizophrenia. Although some methodological issues regarding the tDCS electrode montage or the diagnosis of included patients should be taken into account to explain the observed discrepancies between studies, the methods used to measure BDNF levels also varied between studies. The classical peripheral measure of BDNF (whether in plasma or serum) includes a combination of the three BDNF isoforms that co-exist in the blood: the BDNF precursor protein (proBDNF) and the results of its proteolytic cleavage: the mature BDNF (mBDNF) and the BDNF prodomain [18]. Although the role of BDNF prodomain remains unclear [19], mBDNF and proBDNF exhibit opposite effects on neural plasticity through their binding to specific receptors. ProBDNF preferentially binds to p75 receptors and is involved in LTD mechanisms, pruning of dendritic arborization, cone retractation, and negatively regulates cell survival [20,21,22,23]. Conversely, mBDNF preferentially binds to tropomyosin receptor kinase B (TrkB) receptors and mBDNF-TrkB signaling is involved in LTP mechanisms, survival of neuronal networks, development of dendritic arborization, and neuronal cone growth [24,25,26,27]. The proportion of each isoform may vary depending on internal (such as age, sex, or drugs [28,29,30]) and external factors [24], and on neuronal activity-dependent mechanisms. For instance, low frequency stimulation of cultured hippocampal neurons preferentially induces proBDNF secretion, whereas high-frequency stimulation increases extracellular mBDNF leading to LTP [31]. Despite the opposing implications of the different isoforms on neural plasticity, a large majority of studies in humans measured only total BDNF, whereas one may hypothesize that the lasting beneficial effects observed after tDCS would be supported by a modulation of mBDNF-TrkB signaling involved in LTP mechanisms rather than pathways of other BDNF isoforms.

The aim of this study was therefore to investigate the effects of tDCS on mBDNF levels in patients with schizophrenia. To achieve our goal, we compared mean changes in serum mBDNF levels before and after a single session of either active or sham frontotemporal tDCS in patients with schizophrenia and AVH.

## 2. Materials and Methods

### 2.1. Study Design

This study occurred from 2011 to 2015 at the Centre Hospitalier le Vinatier, psychiatric hospital, Bron, France. In a randomized sham-controlled double-blind trial, participants were randomized to receive either a single session of active or sham tDCS. The study consisted of collecting blood samples once before and once after a single session of tDCS to measure the acute changes in serum mBDNF levels evoked by tDCS. The study was approved by a local ethics committee (Comité de Protection des Personnes Sud-Est VI, France, AU872, 02/02/2011) and complied with international standards for testing with human participants (Declaration of Helsinki). The study was pre-registered in a public database (http://clinicaltrials.gov, registration number NCT02652832, on 12 January 2016). The current study was an ancillary study to a clinical trial previously published elsewhere (NCT00870909; on 27 March 2009) [2]. All participants provided written informed consent before participation.

### 2.2. Participants

**Figure 1:**
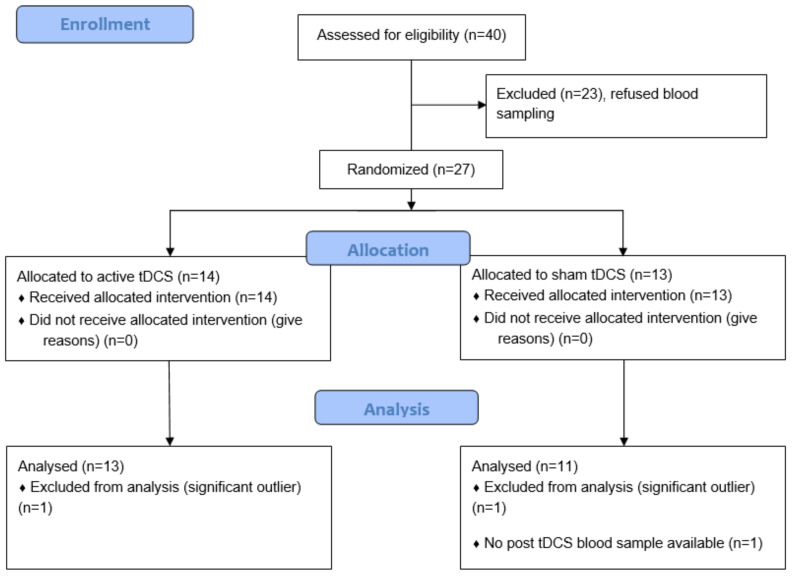
CONSORT flow diagram of the study.

Twenty-seven patients with a DSM-IV-TR diagnosis of schizophrenia agreed to participate in the study. Patients presented with refractory auditory hallucinations, defined as the persistence of daily AVH without remission despite antipsychotic medication at an adequate dosage for at least 3 months and after the failure of at least one other previous treatment with an antipsychotic of different class at adequate dose and duration for the current episode. Exclusion criteria included significant neurological illness; head trauma, history of a seizure not induced by drug withdrawal, current alcohol or drug abuse, or inability to provide informed consent. Since one post-tDCS blood sample was missing, the final sample consisted of data from 26 participants and the analyzed sample from 24 (see Figure 1). Current antipsychotic medication was calculated as chlorpromazine clinically equivalent dose in mg/day following the instructions of Gardner et al. [32].

### 2.3. tDCS Treatment

Stimulation was administered using a DC Plus Stimulator (NeuroConn, GmBH) with an intensity set at 2 mA. The stimulation was delivered during 20 min (ramp-up/ramp-down of 30 s). Two 7 × 5 cm surface electrodes (35 cm^2^) placed in saline-soaked sponges were positioned over the scalp according to the 10/20 placement system for electroencephalogram. The anode was placed over the left prefrontal cortex (midway between F3 and FP1) and the cathode was placed over the left temporo-parietal junction (midway between T3 and P3). Rationale for this montage is justified in a previously published article [2].

The sham stimulation consisted of delivering active stimulation with the same electrode montage but only during the first 40 s of the 20 min period (2 mA, 30 s ramp-up/down). For double-blinding, the study mode of the DC-stimulator was used. It consisted of entering a predefined individual 5-digit code into the tDCS stimulator corresponding to active or sham stimulation, so that both the patient and the tDCS operator were blind to the tDCS condition.

To assess the blinding of participants, four participants were randomly chosen to complete a questionnaire that required them to guess whether they received either active or sham stimulation.

### 2.4. Measures of Serum BDNF

Two blood samples were collected, one before the tDCS session (between 8:00 a.m. and 9:00 a.m.) and one other after the end of the tDCS session (at 11:30 a.m.) on a Monday morning. Since sport practice, tobacco smoking, and alcohol consumption may modulate BDNF levels, participants were asked to avoid physical exercise and alcohol consumption during the 24 h prior to the experiment (Table 1). Tobacco smoking, which is also known to interfere with tDCS-induced aftereffects in patients with schizophrenia [33], was not allowed on the morning of the experiment. Blood samples of 5 mL were collected in fasting participants in a Vacutainer SST™ II Advance tube. After 20 min of clotting time, the whole blood sample was centrifuged at 3500× *g* for 20 min at 4 °C to isolate the serum. The serum was then collected, aliquoted (200 mL), and stored at −24 °C until analysis.

mBDNF levels were quantified by enzyme-linked immunosorbent assay (ELISA), according to the manufacturers’ instructions (mature BDNF Immunoassay, Aviscera Bioscience, Santa-Clara, CA, USA). Serum samples were applied on precoated 96-well plates and allowed to incubate for two hours at room temperature. Plates were successively incubated with anti-human BDNF antibodies, streptavidin-HRP conjugate, and substrate. The reaction was shut down by stop solutions provided by the manufacturer. The absorbance was read at 450 nm with a micro-plate reader (Perkin Elmer Wallac 1420 Victor2, Winpact Scientific Inc, Saratoga, CA, USA). According to a reference curve, mBDNF levels are expressed in pg·mL^−1^. Intertrial reproducibility was controlled with an external standard.

### 2.5. Statistical Analysis

All statistical analyses were performed in Jasp version 0.14. Statistical significance was defined as *p* < 0.05. Baseline demographic and clinical data were compared between groups (active vs. sham) using independent sample *t*-tests for continuous variables and Fischer’s exact tests for categorical variables. The primary outcome was the change in mBDNF levels induced by tDCS (ΔBDNF), calculated as mBDNF level after the tDCS session minus mBDNF level before the tDCS session. Potential outliers were identified with Grubb’s test based on the value of the changes in mBDNF levels (https://www.graphpad.com, accessed on 19 May 2021). To compare the effect of active versus sham tDCS on mBDNF level changes, a one-way ANCOVA was performed with tDCS condition (active vs. sham) as a between-subjects factor and baseline mBDNF levels as a covariate. The choice of introducing baseline mBDNF levels as a covariate was made to reduce possible effects of baseline levels on tDCS-induced changes since the two groups showed a trend toward a significant difference in baseline mBDNF levels (see Section 3). Effect size was estimated using eta squared (η^2^). To investigate the potential influence of other clinical and demographic variables known to have an impact on baseline serum BDNF levels such as age [28], sex [29], illness duration, or medication [30], exploratory Pearson’s correlations were undertaken.

## 3. Results

Two participants were identified as significant outliers regarding our primary outcome, one in each group, and were thus excluded from the analysis. The final analyzed sample consisted of 24 patients: 13 in the active group and 11 in the sham group.

There were no significant differences between the two groups regarding sociodemographic and clinical characteristics (Table 1). However, since baseline mBDNF levels tended to differ between the two groups (*p* = 0.054), we added baseline mBDNF level as a covariate in the analysis to control for this factor. The distribution of the different treatments in terms of dose and molecules between the two groups was relatively balanced and no significant differences were observed (Table 1).

### 3.1. Effect of tDCS on mBDNF Levels

**Figure 2:**
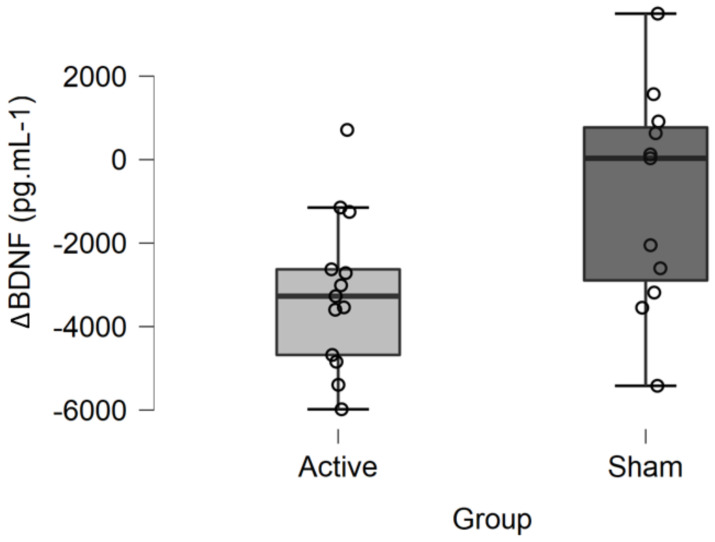
Changes in mBDNF levels (ΔBDNF) after active or sham tDCS. The decrease in serum mBDNF levels was significantly greater after active than after sham tDCS when adjusted for baseline mBDNF levels (F = 5.387; *p* = 0.030; η^2^ = 0.205).

A mean 20% (±14) decrease in mBDNF levels was observed after active tDCS (ΔBDNF = −3181.221 ± 1862 pg·mL^−1^), whereas a mean 8% (±21) decrease was observed after sham tDCS (ΔBDNF = −912.988 ± 2643 pg·mL^−1^) (Figure 2). The ANCOVA revealed a significant main effect of tDCS condition on changes in serum mBDNF levels (F = 5.387; *p* = 0.030; η^2^ = 0.205). In other words, the decrease in serum mBDNF levels was significantly greater after active than after sham tDCS when adjusted for baseline mBDNF levels. There was no significant effect of the covariate baseline mBDNF level on mBDNF changes (F = 0.080, *p* = 0.780, η^2^ = 0.003).

Exploratory Pearson’s correlation analyses undertaken to investigate the influence of clinical and demographic variables on baseline serum mBDNF levels revealed no significant effect of age (*r* = 0.238; *p* = 0.262), dose of antipsychotic medication measured as chlorpromazine equivalent in mg/day (*r* = 0.093; *p* = 0. 666), or illness duration (*r* = 0.178; *p* = 0.404). No significant differences were observed for sex proportion or type of treatments between the two groups at baseline. No difference was observed at baseline between men and women regarding mBDNF levels (men 15,400.287 ± 5126.064 versus women 14569.925 ± 2786.04; *p* = 0.636).

### 3.2. Tolerability and Blinding

All participants tolerated the tDCS session well and no serious adverse events were observed. Only two participants from the sham group reported medium adverse effects after the tDCS session (neck suffering, headache, and concentration difficulties).

Regarding the blinding, all four of the participants who were evaluated belonged to the sham group. Among them, two thought they had received active stimulation, one thought he had received the sham stimulation, and one was not able to guess the condition he received. These results suggest that participants were unable to identify which stimulation they were receiving and that the blinding of participants was well respected.

## 4. Discussion

The aim of the current study was to investigate the effects of a single session of frontotemporal tDCS on serum mBDNF levels in patients with schizophrenia and AVH. Active stimulation led to a significantly greater reduction in mBDNF levels compared with sham stimulation, with a small to medium effect size.

Our findings are consistent with previous studies using other forms of noninvasive brain stimulation (NIBS) techniques that reported a significant decrease in peripheral BDNF levels after stimulation of the prefrontal cortex [34,35,36]. Along this line, it was reported that a single session of repetitive transcranial magnetic stimulation (rTMS) applied over the left prefrontal cortex can decrease BDNF levels in healthy volunteers [34], and that multiple sessions of either low- or high-frequency rTMS applied over the prefrontal cortex can also decrease peripheral BDNF levels in both healthy volunteers [35] and in patients with amyotrophic lateral sclerosis [36]. However, our current findings do not support results from other studies reporting that NIBS did not modulate BDNF levels in patients with depression receiving high frequency rTMS [37], and in patients with uni- or bipolar depression receiving repeated sessions of frontal tDCS with the anode placed over the left prefrontal cortex [15,16,17]. Moreover, the present results are opposite to those from studies reporting that NIBS may induce an increase in BDNF levels in patients with depression receiving high-frequency rTMS over the left prefrontal cortex [38] or in patients with opioid addiction receiving tDCS over the dorsolateral prefrontal cortex [13]. Although all these studies targeted the PFC, the discrepancies between observed results may be related to variations in other NIBS parameters such as the total number of sessions delivered (which varies from 1 to 22 between studies), the type of the NIBS itself (rTMS or tDCS), and the number of sessions delivered by day, all of which are important to take into account [39,40,41,42].

The effects of NIBS on BDNF levels may also be influenced by participant diagnosis, further explaining the heterogeneous results observed in the literature. In line with this hypothesis, low frequency rTMS applied to the prefrontal cortex may induce a decrease in BDNF levels in healthy volunteers but not in patients with amyotrophic lateral sclerosis, who showed a decrease in BDNF levels only after high-frequency rTMS [36]. Thus, NIBS may either increase, decrease, or have no effect on peripheral BDNF levels depending on NIBS parameters and on the diagnosis of participants. To the best of our knowledge, no previous study has investigated the effects of tDCS on BDNF levels in patients with schizophrenia, although some evidence suggests abnormal BDNF regulation in this illness [43,44]. The present findings suggest that a single session of frontotemporal tDCS may decrease mBDNF in patients with schizophrenia and treatment-resistant AVH. Our results cannot be extended to patients with schizophrenia with other types of prominent symptoms (such as those with prominent negative symptoms or those with non-treatment-resistant schizophrenia) or to patients with another psychiatric condition that may have another baseline BDNF status.

The methods used to measure BDNF levels may also contribute to the heterogeneous results observed in the NIBS literature. Firstly, BDNF can be collected from either serum or plasma, while there is no consensus on their relative interpretations. Secondly, to date, the majority of studies have measured the effects of NIBS on total BDNF levels. To the best of our knowledge, the present study is one of the first to specifically explore tDCS effects on mBDNF rather than on total BDNF. Such methodological differences prevent any direct comparison between the current results and the literature. However, one may hypothesize that measuring the effects of one NIBS session on each of the BDNF isoforms is a particularly relevant approach to better understand NIBS-induced neuroplasticity, since proBDNF and mBDNF exhibit opposite effects on plasticity [27]. Due to its beneficial effects on LTP via the BDNF-TrkB signaling pathway, the mBDNF isoform may better reflect the beneficial effects of tDCS on neural plasticity. Nevertheless, BDNF is first synthesized as proBDNF, stored in dense core vesicles in neurons, and thereafter can be cleaved in mBDNF, either in the intracellular or extracellular compartment [45]. Thus, the observed increase, decrease, or null effect of tDCS on total BDNF after tDCS in the literature may be partly explained by compensation phenomena between the isoform concentrations. In this regard, one potential explanation for our results is that reduction in serum mBDNF may reflect an increased use of mBDNF in the central nervous system (CNS) after one tDCS session, resulting in the enhancement in neuronal plasticity induced by the stimulation [11,46].

Additionally, it was reported that BDNF-Val66Met-polymorphism interacts with tDCS dose to predict neurocognitive outcomes in patients with depression [47]. Similarly, BDNF-Val66Met-polymorphism may interact with tDCS to predict cortical plasticity in patients with schizophrenia [48]. Such gene–environment interactions support the hypothesis of a close relationship between tDCS-induced neural plasticity and BDNF, even in patients with schizophrenia.

Finally, we observed a trend toward a statistically significant difference in baseline mBDNF levels between the active and sham groups that may have influenced the present results. To rule out this potentially confounding effect, we introduced baseline mBDNF levels as a covariate in the primary analysis and we undertook exploratory analyses to control for the role of different moderators that may have influenced baseline mBDNF levels in patients with schizophrenia. It was reported that: (i) age negatively correlates with total BDNF levels [28], (ii) men exhibit higher levels of BDNF compared with women in a sample including patients with depression and healthy controls [29], and (iii) antipsychotics may increase BDNF levels in patients with schizophrenia [30] in a dose-dependent manner with clozapine but not with first generation antipsychotics [49]. No differences have been identified between active and sham groups regarding these parameters, and there was no correlation between these parameters and mBDNF levels at baseline. Further analyses did not reveal significant associations between baseline mBDNF and the variables identified above, and no differences were observed between the active and sham groups, suggesting that baseline mBDNF did not influence current results. Another important interaction that may have influenced the results observed in patients with schizophrenia under antipsychotic medication is the close relationship between BDNF synthesis and central dopamine release. It was observed in heterozygous BDNF (BDNF+/−) mice that endogenous BDNF may influence central dopamine neurotransmission by regulating the release and uptake dynamics of pre-synaptic dopamine transmission [50]. The interaction between antipsychotics binding on dopamine receptors, tDCS-induced dopamine release [6], and endogenous levels of BDNF needs further investigation in patients with schizophrenia.

The current study has some limitations that should be acknowledged. Firstly, the present results were obtained from an ancillary study to an RCT investigating the clinical effects of 10 sessions of tDCS on hallucinations in patients with schizophrenia [2]. The current study therefore has a relatively low statistical power, and no a priori sample size calculation was performed regarding the current outcome. Secondly, we investigated here the acute effect of a single session of tDCS on mBDNF, but it would have been interesting to examine the effect of repeated sessions of tDCS on BDNF levels since repeated sessions are usually needed to obtain a sustainable clinical effect. Thirdly, we only measured serum mBDNF levels, which prevented us from investigating the effects of tDCS on other isoforms of BDNF or on total BDNF. Fourthly, we only investigated acute changes after a single session of tDCS, thus no delayed effects of tDCS on mBDNF were investigated. Notably, in animal models, tDCS could either increase or decrease both serum and central BDNF levels depending on the delay between the tDCS sessions and the BDNF measure [51], suggesting that this parameter can be of major interest. Fifthly, while serum BDNF may be a good indicator of BDNF regulation in the central nervous system [52], it may not directly reflect brain fluctuations. However, it was shown that BDNF can cross the blood–brain barrier [53] and that BDNF blood levels correlate with BDNF levels in brain tissues [54]. Finally, we did not investigate the effect of BDNF single nucleotide polymorphism on the response to tDCS, although it was shown that BDNF-Val66Met-polymorphism interacts with tDCS aftereffects [48]. This specific polymorphism possibly interferes with the secretory pathway, resulting in a decreased secretion of BDNF [55,56]. More studies are needed to investigate the effect of this polymorphism on tDCS-induced mBDNF level variation.

## 5. Conclusions

In patients with schizophrenia and AVH, the current study highlighted that one session of active frontotemporal tDCS induces a significantly larger decrease in serum mBDNF levels compared with the sham. Beneficial effects of tDCS observed in patients with schizophrenia may be underpinned by a regulation of the mBDNF-TrkB signaling-related pathway that modulates neural plasticity. This study is a first step toward a better understanding of the mechanisms through which tDCS modulates brain plasticity in patients with schizophrenia. Further studies are needed to explore the effects of tDCS on each BDNF isoform and to investigate the respective roles of BDNF polymorphisms in the mechanisms underlying the physiological effects of tDCS in the CNS.

## Figures and Tables

**Table 1:** Baseline sociodemographic and clinical data of participants in active and sham groups.

	Active Group (Mean ± SD)	Sham Group (Mean ± SD)	*p*-Value
*n* total	13	11	
Age (years)	33.08 ± 8.96	37.18 ± 9.38	0.285
Illness duration (years)	10.38 ± 9.51	14.73 ± 7.79	0.132
Sex (*n*)	6F/7M	5F/6M	0.973
(%)	46%/54%	45%/55%	
Handedness (*n*)	11R/1L/1 both	9R/2L	0.484
Smokers (%)	58%	40%	0.392
Alcohol intake ^1^ (%)	0%	9%	0.267
Physical exercise ^1^ (%)	8%	9%	0.902
PANSS Total	66.00 ± 14.89	69.78 ± 14.89	0.565
PANSS Positive	18.00 ± 4.81	19.44 ± 3.47	0.450
PANSS Negative	19.00 ± 5.63	17.33 ± 5.31	0.493
PANSS General	29.00 ± 5.31	33.00 ± 7.62	0.262
mBDNF (pg·mL^−1^)	16,510.80 ± 4346.98	13,257.50 ± 3274.58	0.054
Antipsychotic dose (CPZeq)	930.41 ± 415.13	1192.91 ± 449.45	0.151
Molecule			
Typical antipsychotics	4	2	0.649
Atypical antipsychotics	12	11	1.000
Clozapine	4	4	1.000
Antidepressants	3	4	0.659
Benzodiazepines	5	2	0.386
Anxiolytics	3	6	0.206

CPZeq, chlorpromazine clinically equivalent dose in mg/day [32]; F, female; M, male; mBDNF, mature brain-derived neurotrophic factor; L, left-hander; PANSS, positive and negative syndrome scale; R, right-hander; SD, standard deviation. ^1^ Alcohol consumption and physical exercise were controlled for the 24 h prior to the experiment. *p*-values were obtained using independent samples *t*-tests for age, PANSS scores, CPZeq and mBDNF levels, and Fischer’s exact test tests for other variables.

## Data Availability

Data will be available upon reasonable request by contacting the corresponding author.
